# Redox Homeostasis and Natural Dietary Compounds: Focusing on Antioxidants of Rice (*Oryza sativa* L.)

**DOI:** 10.3390/nu10111605

**Published:** 2018-11-01

**Authors:** Wiramon Rungratanawanich, Maurizio Memo, Daniela Uberti

**Affiliations:** Department of Molecular and Translational Medicine, University of Brescia, 25123 Brescia, Italy; w.rungratanawani@unibs.it (W.R.); maurizio.memo@unibs.it (M.M.)

**Keywords:** natural compound, phytochemical, rice, *Oryza sativa* L., γ-oryzanol, redox homeostasis, Nrf2, oxidative stress, ROS, antioxidant

## Abstract

Redox homeostasis may be defined as the dynamic equilibrium between electrophiles and nucleophiles to maintain the optimum redox steady state. This mechanism involves complex reactions, including nuclear factor erythroid 2-related factor 2 (Nrf2) pathway, activated by oxidative stress in order to restore the redox balance. The ability to maintain the optimal redox homeostasis is fundamental for preserving physiological functions and preventing phenotypic shift toward pathological conditions. Here, we reviewed mechanisms involved in redox homeostasis and how certain natural compounds regulate the nucleophilic tone. In addition, we focused on the antioxidant properties of rice and particularly on its bioactive compound, γ-oryzanol. It is well known that γ-oryzanol exerts a variety of beneficial effects mediated by its antioxidant properties. Recently, γ-oryzanol was also found as a Nrf2 inducer, resulting in nucleophilic tone regulation and making rice a para-hormetic food.

## 1. Introduction

Redox homeostasis may be defined as the internal dynamic equilibrium with respect to the continuous alterations of electrophilic and nucleophilic tone in order to maintain the optimum redox steady state [1,2,3]. The maintenance of redox homeostasis is crucial for preserving physiological functions since reactive oxygen and nitrogen species (ROS/RNS) are constantly generated in the normal metabolism of aerobic cells [4]. In fact, redox homeostasis alterations, as the result of the inability to maintain the optimal redox steady state, are associated with a phenotypic shift toward pathological conditions [5,6]. According with the Harman theory of aging, an imbalance between the efficiency of antioxidant systems and the production of free radicals, derived from mitochondrial respiration and/or external environmental stressors, is at the base of aging process alterations and the development of age-related diseases. Following this concept, scavenging free radicals from a cellular environment might be the right approach for a beneficial effect. Indeed, ROS/RNS are not intrinsically harmful, and the Harman theory today could be too simplistic [7]. The fine regulation of the dynamic equilibrium between electrophilic and nucleophilic tone is at the base of redox homeostasis. When electrophilic tone increases, nucleophilic feedback reactions will be activated through the engagement of redox signaling in order to restore the system back to the initial steady state [5,8]. These reactions involve the antioxidant response element (ARE or also known as electrophile response element, EpRE)-regulated phase II enzymes such as NAD(P)H: quinone oxidoreductase 1 (NQO1), heme oxygenase-1 (HO-1), and glutathione *S*-transferase (GST), which transcriptional activation requires a basic leucine zipper transcription factor called nuclear factor erythroid 2-related factor 2 (Nrf2) [9,10,11]. 

Natural antioxidants have recently obtained great attention in preventing lifestyle and age-related diseases. They contain phytochemicals that help the body to counteract oxidative stress by directly scavenging free radicals or activating antioxidant pathways [12,13,14,15]. Recently, new evidence has shown that certain natural compounds are able to induce ARE-regulated phase II enzymes, thus participating in the maintenance of redox homeostasis [16,17,18,19,20,21,22,23,24,25,26,27,28,29,30]. A good understanding of the mechanisms by which certain natural compounds preserve nucleophilic tone is of great interest in the field of the public health prevention, especially in the aging population.

Thus, the aim of this review is to summarize the mechanisms involved in redox homeostasis and how certain natural compounds regulate the nucleophilic tone. In addition, we discussed on the antioxidant properties of rice focusing on γ-oryzanol, underlining the relationship between chemical structures and biological effects. Besides, based on our recent finding, we emphasized a possible role of γ-oryzanol as a para-hormetic natural compound.

## 2. Oxidants and the Electrophilic Tone Regulation

Since 1985, the concept of oxidative stress has been introduced to redox biological research [31]. In general, the term of oxidative stress is referred to an imbalance between the efficiency of antioxidant capacity and free radical production. Several types of reactive species are generated in the body in the form of free radicals or non-radicals as the result of normal metabolic reactions or the exposure to exogenous toxins and pathological events. These species include oxygen derivatives (i.e., O_2_^∙−^, OH^∙^, OH_2_^−^, H_2_O_2_) and nitrogen derivatives (i.e., NO, NO_2_, N_2_O_3_, ONOO^−^) [32,33,34,35,36]. Besides the mitochondria [37,38], ROS can be formed by enzymes using molecular oxygen such as cytochrome P450, xanthine oxidase, and plasma membrane-bound NADPH oxidase [39,40]. RNS are synthesized by nitric oxide synthase generating nitric oxide, which can react with superoxide forming a potent oxidant peroxynitrite. This peroxynitrite can further react with other molecules to produce more RNS such as nitrogen dioxide and dinitrogen trioxide [39,40].

Although it is widely believed that these free radicals and oxidized products can cause cellular and tissue damage, it is also true that the nature has selected ROS/RNS as a signal transduction mechanism responsive to the effects of nutrients and oxidative environment. Only in the past two decades, it has become apparent that ROS serve as signaling molecules to regulate biological and physiological processes [32,33,34,40,41,42,43]. Their roles as harmful compounds or signal molecules depend on the types of ROS/RNS, duration of the stimulus, and their local concentrations [35,44]. Redox signaling involves specific electrophiles that react with specific protein thiolates, and this redox shift is rapidly reverted by feedback reactions. In this context, redox post-translational modifications (rPTMs) of cysteine residues, reminiscent of phosphorylation and ubiquitination of critical amino acids, regulate a broad spectrum of protein activities [45]. For example, in redox signaling pathway, thiolate anion (Cys-S) of cysteine residues can be oxidized by H_2_O_2_ to sulfenic (Cys-SOH), modulating protein functions. This reaction can be reverted by thioredoxin and glutaredoxin, thus ensuring the fine regulation of signal transduction. On the other hand, accumulation of H_2_O_2_ can further oxidize sulfenic (Cys-SOH) to sulfinic (Cys-SO_2_H) and sulfonic (Cys-SO_3_H) species, both of which are irreversible mechanisms and permanently damage protein structures and functions [46,47]. RNS are also involved in redox signaling pathway through *S*-nitrosylation, generating *S*-nitrosoproteins [48]. *S*-nitrosylation is the reversible reaction of nitric oxide-derived species with thiols of cysteine residues through oxidation or transnitrosylation, a transferring of NO [45,49]. The relevance of *S*-nitrosylation role as a pleiotropic player of protein rPTMs is underlined by the specificity of target substrates and the enzymatic mechanisms involved in *S*-nitrosylation/denitrosylation [45,49]. *S*-nitrosylation regulates diverse pathways such as G-protein-coupled receptor signaling [50], glutamate-dependent neurotransmission [51], vesicular trafficking [52], death receptor-mediated apoptosis [53], and stimulation of prostaglandin synthesis [54]. On the other hand, aberrant *S*-nitrosylation affects protein functions, and it is related to the development of various diseases including cancer, diabetes type 1 and 2, cardiovascular (CVD), Parkinson’s (PD), and Alzheimer’s (AD) diseases [55]. Another example is the lipid peroxidation, the reaction of oxidative chain degradation in lipid cell membrane. This reaction is activated by free radicals in polyunsaturated fatty acids of lipid membrane, producing lipid hydroperoxides (LOOH) and their derivatives [56,57]. LOOH are further catalyzed by lipoxygenases enzyme, which also requires the activation of hydroperoxide, resulting in cellular damage [58,59]. However, this chain reaction of lipid peroxidation also produces one of α, β-unsaturated aldehydes called 4-hydroxynonenal (HNE). HNE is mainly generated from the reaction between n-6 fatty acids and hydroperoxide, and functions as a signal transduction molecule for redox homeostasis [59,60,61,62]. In normal state, HNE is produced in the proper amount to maintain redox steady state modulating the mRNA expression of antioxidant enzymes [63]. While in the presence of oxidative stress, the production of HNE is substantially increased and able to act as a signaling species for nucleophilic feedback to counteract oxidative stress and maintain the original steady state [64,65]

## 3. Antioxidants and the Maintenance of Neutrophilic Tone

To counteract the effects of electrophiles and oxidants, the body is endowed with a category of compounds called antioxidants. These antioxidants are produced endogenously (i.e., enzymes: superoxide dismutase (SOD), catalase (CAT), and glutathione peroxidase (GPx)) or received from exogenous sources (i.e., vitamins: vitamin C and E; minerals: zinc (Zn), manganese (Mn), and selenium (Se); and phenolic compounds). They represent the first defense against a burst of ROS/RNS to restore nucleophilic tone. Antioxidant enzyme cascade reacts sequentially to neutralize free radicals. SOD catalyzes the dismutation of superoxide anion producing H_2_O_2_, which is in turn decomposed by CAT or GPx to water [66,67,68,69]. Vitamins, phenolic compounds, and minerals are not endogenously produced, thus they have to be integrated from diet. Vitamin C is a hydrosoluble, while vitamin E is a liposoluble antioxidants [70,71,72]. Also, phenolic compounds are one of the most common antioxidants found in food [73,74,75]. Thanks to their chemical structures, these vitamins and phenolic compounds can neutralize free radicals by donating hydrogen atoms and become stable resonance structures, thereby protecting cell membranes and proteins from oxidative damage [75,76,77,78,79,80,81]. Dietary minerals are known as essential cofactors of antioxidant enzymes involved in redox homeostasis. For example, Zn, Mn, and Se function in various classes of enzymes. Zn is present in cytosolic SOD (SOD1). Mn is well-known for mitochondrial SOD (SOD2), while Se is a co-enzyme of GPx [82,83,84].

In addition, cellular stress response-related enzymes, also called ARE-regulated phase II enzymes (HO-1, NQO1, and GST), are engaged in long-lasting maintenance of the redox homeostasis, supporting the nucleophilic tone [10,11]. These enzymes are transcriptionally under the control of Nrf2 through its binding to the consensus ARE within the 5′-flanking promoter region of these target genes. Nrf2 is considered as a sentinel of oxidative stress and protects the body by making it more resistant to oxidative insults [85]. For instance, Nrf2 knockout mice are substantially more susceptible to a broad range of chemical toxicity and disease conditions associated with oxidative pathology such as AD, PD, and amyotrophic lateral sclerosis (ALS) [86,87,88,89,90,91,92,93]. Activation of Nrf2-mediated gene transcription involves various complex processes [94,95]. Nrf2 generally binds to Kelch like ECH associated protein 1 (Keap1) in the cytoplasm. Keap1 is a protein regulator playing a key role in controlling the steady state of Nrf2 pathway based on redox conditions. In basal or unstressed conditions, Nrf2 is less activated and has rapid turnover rate with short half-life due to the formation of Nrf2-Keap1-Cul3 complex in the cytoplasm. Keap1, which interacts with Cul3-E3-ligase (an ubiquitin ligase), binds to Nrf2 and helps in promoting Nrf2 ubiquitination leading to rapid proteasomal degradation by 26 S proteasome [96,97,98,99,100,101,102,103]. This complex can be interrupted by various electrophiles resulting in the activation of Nrf2 signaling pathway. In the presence of oxidative stress, Keap1 further functions as a stress signal through the stress-induced oxidation of its key cysteine residues. The stimuli can oxidize or covalently modify disulfide bond (-S-S-) of cysteine residues causing conformational changes and interrupt the Keap1-Cul3 complex by inhibiting the ubiquitin E3 ligase activity. The reaction decreases the ability of Keap1 to bind Nrf2, thereby freeing Nrf2 and activating its nuclear translocation. Before its nuclear translocation, Nrf2 is phosphorylated by protein kinases (protein kinase C-δ (PKCδ) and protein kinase B (Akt)), which are also induced by electrophiles and oxidants. When Nrf2 is translocated into the nucleus, it dimerizes with the small Maf heterodimer proteins and binds to a cis-acting element of ARE activating the transcription of ARE-dependent phase II enzymes such as HO-1, GST, and NQO1 [96,99,103,104,105,106,107,108]. In addition, this Nrf2-ARE activation is also found to control the expression of several cytoprotective genes such as thioredoxin 1, thioredoxin reductase 1, sulfiredoxin 1, NADPH-generating enzymes (glucose-6-phosphate dehydrogenase (*G6PD*), 6-phosphogluconate dehydrogenase (*PGD*), malic enzyme (*ME*)1, and isocitrate dehydrogenase (*IDH*)1), ferritin, and glutathione-based system (*GPx*, glutathione disulfide (*GSSG*), glutathione reductase (*GSR*)) [105,109,110,111,112,113,114,115,116,117,118,119,120].

Interestingly, several natural antioxidant compounds, thanks to their electrophilic properties, are Nrf2-ARE inducers. Most of them consist of oxidizable phenols, quinones, Michael acceptors (olefins), isothiocyanates, dithiolethiones, vicinal dimercaptans, or polyenes [121,122,123,124,125,126,127]. These antioxidant compounds and their oxidized derivatives have the ability to react (by oxidation, reduction, and alkylation) with sulfhydryl group of cysteine residues of Keap1, favoring Nrf2 nuclear translocation and activation of ARE signaling process [128,129,130,131,132,133,134,135,136]. It is noteworthy that chemical structure of HNE (α, β-unsaturated aldehyde), one of the most effective endogenous Nrf2 activator, is also a common functional group found in many natural antioxidants [137,138]. Thus, the antioxidant potential of natural products may stem from mimicking the signaling of endogenous electrophiles by activating Nrf2 to restore nucleophilic tone and maintain the equilibrium of redox homeostasis. This mechanism is called “para-hormesis” [96].

## 4. Rice Antioxidants

Rice has been a primary staple food for billions of people worldwide and also represents cultural identity and global unity for centuries [139,140]. Rice that is cultivated for consumption has two major species: *Oryza sativa* and *Oryza glaberrima. Oryza sativa* species are the varieties originated from South-East Asia and also found throughout Asia, America, and Europe. *Oryza glaberrima* species are originated from West Africa and only grown in this area [141]. Rice from all the varieties can be further categorized into two subspecies, *Japonica* and *Indica*, based on the degrees of spikelet and pollen sterility in F1 hybrids between them. Rice contains essential amino acids, dietary fibers, carotenoids, folate, lignin, and minerals, and it is rich in many bioactive phytochemicals: γ-oryzanol, vitamin E (tocopherols and tocotrienols), γ-aminobutyric acid (GABA), phenolics, flavones, and anthocyanins [142,143,144,145]. Studies in rice have shown that rice is not only important in terms of a staple food, but also play a role in promoting various health benefits such as anti-inflammation, anti-diabetic, anti-hyperlipidemic, anti-cancer, and antioxidant potential [146,147,148,149,150,151,152,153,154].

**Figure 1:**
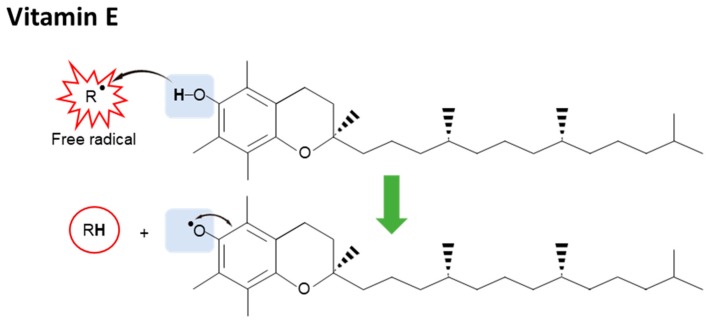
Antioxidants (vitamin E) in rice and structure-activity relationship. Vitamin E exerts its antioxidant effects by quenching free radicals. The hydroxyl group on chromanol ring can donate its hydrogen atom to a free radical resulting in a delocalized and stabilized unpaired electron, vitamin E radical.

Some of the therapeutic effects of rice in preventing diabetes type 2 (T2D), CVD, obesity, different types of cancer, and inflammation are attributed to its antioxidant properties [155,156,157,158]. The antioxidant properties of rice have been first published in 1989, which some of its active compounds including flavonoids, α-tocopherols, and γ-oryzanol were identified [159,160]. Since 2000, research interest on rice has been improved and the number of research articles related to its antioxidant properties dramatically increased more than 15 times [161,162]. Today, it is known that rice contains about 100 kinds of antioxidants, which can be essentially classified into two major groups: vitamins and phenolic–based compounds [163,164,165]. The mechanisms by which these bioactive compounds exert antioxidant activity have been investigated [166,167]. For example, it is well known that vitamin E exerts its antioxidant effects by quenching free radicals. Vitamin E in rice includes tocopherols (α,β,γ,δ forms) and tocotrienols (α,β,γ,δ forms) [143]. Thanks to its structure of the chromanol ring connected with a free hydroxyl group, hydrogen atom of the hydroxyl group can be donated to a free radical resulting in the delocalized and stabilized unpaired electron, vitamin E radical (Figure 1). Since the reactivity of the vitamin E radical is much less than other radicals, it is relatively stable to break the radical chain cascade [168,169,170,171].

**Figure 2:**
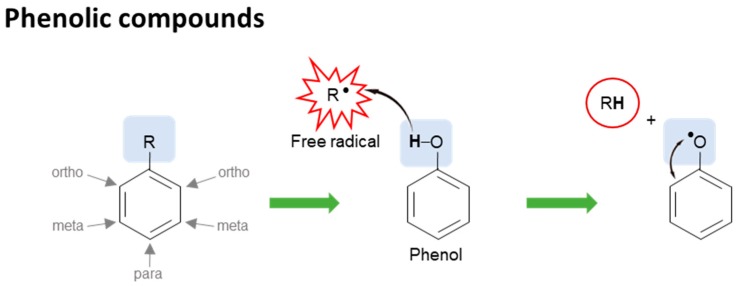
Antioxidants (phenolic compounds) in rice and structure-activity relationship. Phenolic compounds act as free radical scavengers since hydroxyl group on the phenolic ring can transfer its hydrogen atom to a free radical, forming a delocalized and stabilized unpaired electron, phenoxy radical, across the phenolic ring. The antioxidant capacity of phenolic compounds is in function of the numbers of hydroxyl groups, the location of hydroxyl group on aromatic ring (*ortho*, *para*, *meta* positions), and the presence of other functional groups on the molecule.

Phenolic compounds consist of at least one aromatic ring and one hydroxyl group [172,173]. This chemical structure is present in many active compounds including ferulic, cinnamic, *p*-coumaric, caffeic, sinapic, chlorogenic, gallic, vanillic, *p*-hydroxybenzoic, protocatechuic, and syringic acids, flavonoids, and their derivatives [163,164,165]. Those found more in pigmented rice are anthocyanins (cyanidin-3-*O*-glucoside, cyanidin-3-*O*-galactoside, cyanidin-3-*O*-rutinoside, peonidin-3-*O*-glucoside, and pelargonidin-3-*O*-glucoside) and proanthocyanidins, which are responsible for purple to black color and reddish color respectively, while the ferulic acid esters are more abundant in rice bran layer [174,175,176,177,178,179]. The phenolic-based structure highly provides antioxidant properties as free radical scavengers. The hydroxyl group on the phenolic ring can transfer its hydrogen atom to a free radical forming a delocalized and stabilized unpaired electron, phenoxy radical, across the phenolic ring (Figure 2). The antioxidant capacity of phenolic-based compounds is in function of the numbers of hydroxyl groups, the location of hydroxyl group on aromatic ring (ortho, para, meta positions), and the presence of other functional groups on the molecule [180,181]. The antioxidant activities of these compounds are four times stronger than that of α-tocopherol [182,183,184]. In addition, some of these compounds such as anthocyanins and ferulic acid derivatives are also able to activate ARE-regulated phase II enzyme expression [185,186,187,188,189].

## 5. Γ-Oryzanol: Structure-Antioxidant Activity Relationship

Γ-Oryzanol is present in the highest amount in rice as a mixture of phytosteryl ferulates containing ferulic acid esters of phytosterols (sterols and triterpene alcohols) [55,190]. In γ-oryzanol, there are at least 10 different phytosteryl ferulates such as cycloartenyl ferulate, 24-methylenecycloartanyl ferulate, campesteryl ferulate, sitosteryl ferulate, stigamsteryl ferulate, campestanyl ferulate, sitostanyl ferulate, Δ7-campestenyl ferulate, Δ7-sitostenyl ferulate, and Δ7-stigmastenyl ferulate [55,190,191,192]. Among these, the principal components accounting for approximately 80% are cycloartenyl, 24-methylenecycloartanyl, campesteryl, and sitosteryl ferulates [193,194,195,196,197].

Γ-Oryzanol has been proposed as a potent antioxidant compound [198]. A growing number of studies have demonstrated that γ-oryzanol acts as a free radical scavenger and improves the activity of endogenous antioxidant enzymes. Γ-Oryzanol major components (campesteryl ferulate, cycloartenyl ferulate, and 24-methylenecycloartenyl ferulate) are more potent antioxidants than all components of vitamin E, and 24-methylenecycloartenyl ferulate has the highest antioxidant activity [199]. The antioxidant properties of γ-oryzanol tested with different types of radicals—such as inorganic oxygen-derived radicals, DPPH radicals, lipid soluble organic radicals, 2,2′-azinobis-3-ethylbenzothiazoline-6-sulfonic acid (ABTS) free radicals, and linoleic acid peroxidation—revealed that γ-oryzanol acts as organic, lipophilic as well as hydrophilic radical scavengers [200,201]. Moreover, γ-oryzanol was found to possess SOD-like activity in inhibiting superoxide radical catalyzed pyrogallol autoxidation [202]. In *in vitro* cells, pretreatment with the main compounds of γ-oryzanol (sitosteryl ferulate, cycloartenyl ferulate, and 24-methylenecycloartenyl ferulate) prevented H_2_O_2_-induced ROS production via scavenging free radicals [203]. Likewise, we recently demonstrated in HEK-293 cells that γ-oryzanol pretreatment prevents H_2_O_2_-induced ROS generation by increasing the activity and protein expression of antioxidant enzymes such as SODs [204].

The antioxidant properties of γ-oryzanol have also been shown in *in vivo* models. In *Drosophila melanogaster* model, γ-oryzanol enhanced antioxidant defense by significantly improving the antioxidant enzymes such as SOD, CAT, and GST, and decreasing the malondialdehyde (MDA) and ROS production [205]. In streptozotocin-induced oxidative stress, γ-oryzanol also effectively increased the levels of SOD and reduced glutathione in rats [206]. In mice model of ethanol-induced liver injury, γ-oryzanol was able to prevent increased hepatic lipid hydroperoxide, TBARS levels as well as plasma aspartate and alanine aminotransferase activities. Moreover, in the same study, γ-oryzanol also improved SOD activity, suggesting that its effects in preventing ethanol-induced hepatic injury could be mediated by its antioxidant properties [207]. Interestingly, the effects of γ-oryzanol were also evaluated in animals fed with high fat diet (HFD). What the authors found was a significant increase of antioxidant enzyme activities and decrease of free radicals in those mice fed with HFD supplemented with γ-oryzanol compared with control HFD animals. Furthermore, in these mice, γ-oryzanol decreased hepatic lipogenesis and suppressed plasma triglyceride and total cholesterol levels with an increase of HDL cholesterol concentrations [208]. The effects on lipid metabolism of γ-oryzanol were also investigated in dyslipidemic patients, where its beneficial effects were compared with other different dietary supplements including vitamin E and polyunsaturated fatty acids (PUFA) n-3. Among these, the group consuming γ-oryzanol expressed a greater lowering of oxidative stress via regulation of ROS levels, total antioxidant capacity, and inflammatory biomarkers—i.e., tumor necrosis factor (TNF-a), interleukin-1β (IL-1β), and thromboxane B2 (TXB2)—supporting the notion that the anti-hyperlipidemic effects of γ-oryzanol are mediated by its antioxidant properties [209].

The peculiar beneficial properties of γ-oryzanol lay also on its ability to regulate the transcriptional expression of genes related to redox homeostasis and cell survival. For example, in SH-SY5Y cells challenged with H_2_O_2_, γ-oryzanol decreased oxidative stress and prevented neurotoxicity by upregulating antioxidant genes (*SODs)* and anti-apoptotic genes (*NF-κB* and *Bcl-2)* and by downregulating pro-apoptotic genes (*TNF*, *BAX*, and caspase-9) [13]. Recently, we further demonstrated that γ-oryzanol activated Nrf2 nuclear translocation and Nrf2-ARE pathway, with an increase of mRNA and protein expression of ARE-response phase II enzymes such as HO-1, NQO1 [204]. The activation of Nrf2 pathway seems to be central in the biological effects of γ-oryzanol. In fact, various of those aforementioned genes are found to be directly or indirectly regulated by Nrf2. For example, Niture and Jaiswal [210], by using band/supershift and ChIP assays, showed a direct interaction between Nrf2 and Bcl-2 antioxidant response element leading to activation of *Bcl-2* gene expression. NF-κB and TNF are not directly under the control of Nrf2, but it could be modulated by target genes of Nrf2 such as *HO-1* and *NQO1* [211,212]. Likewise, Nrf2 was found also to regulate the expression of *BAX* and caspase-9 in human glioblastoma cells [213].

**Figure 3:**
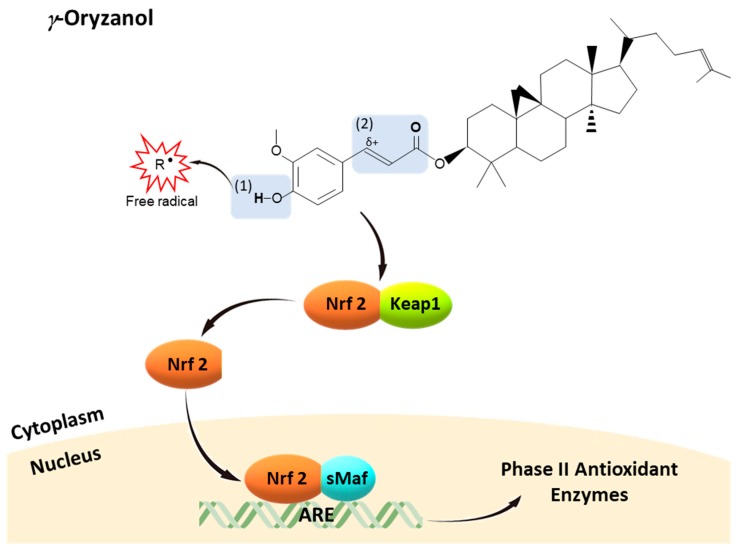
Antioxidants (γ-oryzanol) in rice and structure-activity relationship. (1) The 4-hydroxyl group on the phenolic ring is responsible for hydrogen atom transfer reaction, the hydroxyl group forms a phenoxy radical by transferring its hydrogen atom to a free radical, which contributes to the free radical scavenging properties. This phenoxy radical is highly resonance stabilized because the unpaired electron is able to delocalize across the oxygen to the phenolic ring and α, β-unsaturated carbonyl moiety. (2) The presence of α, β-unsaturated carbonyl moiety of γ-oryzanol is responsible for its electrophilicity. This carbonyl group can delocalize an electron across the oxygen to the olefin, inducing a partial cation (positive charge) at the (olefin) carbon atom, thereby providing this carbon atom an electrophilicity to attract electrons and nucleophiles particularly cysteine residues of Keap1, leading to Nrf2 nuclear translocation and in turn Nrf2-ARE activation.

Therefore, from a structure-activity point of view, (Figure 3) γ-oryzanol could possess free radical scavenging properties due to the transfer of hydrogen atom of 4-hydroxyl group on the phenolic ring of ferulic acid moiety, forming a phenoxy radical. This phenoxy radical is highly stabilized by the delocalization of the unpaired electron across the phenolic ring, unsaturated side chain, and carbonyl group (α, β-unsaturated carbonyl moiety) [214,215,216,217]. On the other hand, γ-oryzanol might activate Nrf2 pathway through at least two possible mechanisms: (1) the formation of more stable oxygen species including H_2_O_2_ during the transferring of hydrogen atoms to free radical species, which could be a signaling molecule for Nrf2 activation [182,218,219,220]; (2) the presence of α, β-unsaturated carbonyl moiety is responsible for its electrophilicity, a main common property of Keap1-Nrf2-ARE pathway inducers. α, β-Unsaturated carbonyl moiety, a Michael acceptor, is a carbon–carbon double bond (olefin) conjugated with an electron-withdrawal carbonyl group [123,221]. This carbonyl group can delocalize an electron across the oxygen to the olefin inducing a partial cation (positive charge) at the (olefin) carbon atom, thereby providing this carbon atom an electrophilicity. Thus, α, β-unsaturated carbonyl moiety becomes an electrophilic moiety to attract electrons and nucleophiles, particularly cysteine residues of Keap1 leading to oxidation of Keap1, Nrf2 nuclear translocation, and in turn Nrf2-ARE activation [222,223] Interestingly, the α, β-unsaturated carbonyl moiety in γ-oryzanol is structurally similar to α, β-unsaturated aldehyde of HNE, an endogenous electrophile signaling molecule known as a Nrf2 inducer [123,221]. Therefore, γ-oryzanol possesses peculiar chemical properties, from both ferulic acid moiety and phytosterols, to counteract oxidative stress and mimic body electrophiles to activate nucleophilic feedback reactions, restoring redox homeostasis.

## 6. Concluding Remarks

The concept of food and its bioactive nutrients to maintain nucleophilic tone restoring redox homeostasis is of great interest in preventing pathology and even in curing chronic diseases. The fact that these natural compounds, including γ-oryzanol, have been demonstrated as Nrf2 inducers provides new insights into their mechanisms of actions supporting and encouraging their use. Indeed, γ-oryzanol is already used as a prescription drug in Japan to treat various conditions such as hyperlipidemia, irritable bowel syndrome, autonomic ataxia, and menopausal syndrome [224,225,226]. Γ-Oryzanol is certainly a promising natural dietary compound, being present in high amounts in the principal grain of human diet.

## References

[1] Medzhitov R. (2008). Origin and physiological roles of inflammation. Nature.

[2] Sen C.K., Packer L. (1996). Antioxidant and redox regulation of gene transcription. FASEB J..

[3] Mattson M.P. (2008). Hormesis defined. Ageing Res. Rev..

[4] McCord J.M. (2000). The evolution of free radicals and oxidative stress. Am. J. Med..

[5] Brigelius-Flohe R., Flohe L. (2011). Basic principles and emerging concepts in the redox control of transcription factors. Antioxid. Redox Signal..

[6] Foyer C.H., Noctor G. (2005). Redox homeostasis and antioxidant signaling: A metabolic interface between stress perception and physiological responses. Plant Cell.

[7] Harman D. (1956). Aging: A theory based on free radical and radiation chemistry. J. Gerontol..

[8] Cadenas E., Sies H. (1985). Oxidative stress: Excited oxygen species and enzyme activity. Adv. Enzyme Regul..

[9] Itoh K., Chiba T., Takahashi S., Ishii T., Igarashi K., Katoh Y., Oyake T., Hayashi N., Satoh K., Hatayama I. (1997). An nrf2/small maf heterodimer mediates the induction of phase ii detoxifying enzyme genes through antioxidant response elements. Biochem. Biophys. Res. Commun..

[10] Son T.G., Camandola S., Mattson M.P. (2008). Hormetic dietary phytochemicals. Neuromol. Med..

[11] Maher J., Yamamoto M. (2010). The rise of antioxidant signaling—The evolution and hormetic actions of nrf2. Toxicol. Appl. Pharmacol..

[12] Lu M.C., Ji J.A., Jiang Z.Y., You Q.D. (2016). The keap1-nrf2-are pathway as a potential preventive and therapeutic target: An update. Med. Res. Rev..

[13] Ismail N., Ismail M., Imam M.U., Azmi N.H., Fathy S.F., Foo J.B., Abu Bakar M.F. (2014). Mechanistic basis for protection of differentiated sh-sy5y cells by oryzanol-rich fraction against hydrogen peroxide-induced neurotoxicity. BMC Complement. Altern. Med..

[14] Ismail N., Ismail M., Fathy S.F., Musa S.N., Imam M.U., Foo J.B., Iqbal S. (2012). Neuroprotective effects of germinated brown rice against hydrogen peroxide induced cell death in human sh-sy5y cells. Int. J. Mol. Sci..

[15] Islam M.S., Nagasaka R., Ohara K., Hosoya T., Ozaki H., Ushio H., Hori M. (2011). Biological abilities of rice bran-derived antioxidant phytochemicals for medical therapy. Curr. Top. Med. Chem..

[16] Granado-Serrano A.B., Martin M.A., Bravo L., Goya L., Ramos S. (2012). Quercetin modulates nrf2 and glutathione-related defenses in hepg2 cells: Involvement of p38. Chem. Biol. Interact..

[17] Lin W., Wu R.T., Wu T., Khor T.O., Wang H., Kong A.N. (2008). Sulforaphane suppressed lps-induced inflammation in mouse peritoneal macrophages through nrf2 dependent pathway. Biochem. Pharmacol..

[18] Farombi E.O., Shrotriya S., Na H.K., Kim S.H., Surh Y.J. (2008). Curcumin attenuates dimethylnitrosamine-induced liver injury in rats through nrf2-mediated induction of heme oxygenase-1. Food Chem. Toxicol..

[19] Jagatha B., Mythri R.B., Vali S., Bharath M.M. (2008). Curcumin treatment alleviates the effects of glutathione depletion in vitro and in vivo: Therapeutic implications for parkinson’s disease explained via in silico studies. Free Radic. Biol. Med..

[20] Balogun E., Hoque M., Gong P., Killeen E., Green C.J., Foresti R., Alam J., Motterlini R. (2003). Curcumin activates the haem oxygenase-1 gene via regulation of nrf2 and the antioxidant-responsive element. Biochem. J..

[21] Rubiolo J.A., Mithieux G., Vega F.V. (2008). Resveratrol protects primary rat hepatocytes against oxidative stress damage: Activation of the nrf2 transcription factor and augmented activities of antioxidant enzymes. Eur. J. Pharmacol..

[22] Ho C.-Y., Cheng Y.-T., Chau C.-F., Yen G.-C. (2012). Effect of diallyl sulfide on in vitro and in vivo nrf2-mediated pulmonic antioxidant enzyme expression via activation erk/p38 signaling pathway. J. Agric. Food Chem..

[23] Korenori Y., Tanigawa S., Kumamoto T., Qin S., Daikoku Y., Miyamori K., Nagai M., Hou D.X. (2013). Modulation of nrf2/keap1 system by wasabi 6-methylthiohexyl isothiocyanate in are-mediated nqo1 expression. Mol. Nutr. Food Res..

[24] Wu K.C., McDonald P.R., Liu J., Klaassen C.D. (2014). Screening of natural compounds as activators of the keap1-nrf2 pathway. Planta Med..

[25] Park J.H., Choi J.W., Ju E.J., Pae A.N., Park K.D. (2015). Antioxidant and anti-inflammatory activities of a natural compound, shizukahenriol, through nrf2 activation. Molecules.

[26] Lan X., Han X., Li Q., Wang J. (2017). (−)-epicatechin, a natural flavonoid compound, protects astrocytes against hemoglobin toxicity via nrf2 and ap-1 signaling pathways. Mol. Neurobiol..

[27] Hajra S., Basu A., Singha Roy S., Patra A.R., Bhattacharya S. (2017). Attenuation of doxorubicin-induced cardiotoxicity and genotoxicity by an indole-based natural compound 3,3’-diindolylmethane (dim) through activation of nrf2/are signaling pathways and inhibiting apoptosis. Free Radic. Res..

[28] Sathibabu Uddandrao V.V., Brahmanaidu P., Nivedha P.R., Vadivukkarasi S., Saravanan G. (2018). Beneficial role of some natural products to attenuate the diabetic cardiomyopathy through nrf2 pathway in cell culture and animal models. Cardiovasc. Toxicol..

[29] Guo C., Wang S., Duan J., Jia N., Zhu Y., Ding Y., Guan Y., Wei G., Yin Y., Xi M. (2017). Protocatechualdehyde protects against cerebral ischemia-reperfusion-induced oxidative injury via protein kinase cepsilon/nrf2/ho-1 pathway. Mol. Neurobiol..

[30] Ali T., Kim T., Rehman S.U., Khan M.S., Amin F.U., Khan M., Ikram M., Kim M.O. (2018). Natural dietary supplementation of anthocyanins via pi3k/akt/nrf2/ho-1 pathways mitigate oxidative stress, neurodegeneration, and memory impairment in a mouse model of alzheimer’s disease. Mol. Neurobiol..

[31] Sies H. (2015). Oxidative stress: A concept in redox biology and medicine. Redox Biol..

[32] Cross C.E., Halliwell B., Borish E.T., Pryor W.A., Ames B.N., Saul R.L., McCord J.M., Harman D. (1987). Oxygen radicals and human disease. Ann. Intern. Med..

[33] Finkel T. (2011). Signal transduction by reactive oxygen species. J. Cell Biol..

[34] Poljsak B., Suput D., Milisav I. (2013). Achieving the balance between ros and antioxidants: When to use the synthetic antioxidants. Oxid. Med. Cell. Longev..

[35] Fridovich I. (1997). Superoxide anion radical (O_2_^−^·), superoxide dismutases, and related matters. J. Biol. Chem..

[36] Kohen R., Nyska A. (2002). Oxidation of biological systems: Oxidative stress phenomena, antioxidants, redox reactions, and methods for their quantification. Toxicol. Pathol..

[37] Forman H.J., Azzi A. (1997). On the virtual existence of superoxide anions in mitochondria: Thoughts regarding its role in pathophysiology. FASEB J..

[38] Brand M.D. (2010). The sites and topology of mitochondrial superoxide production. Exp. Gerontol..

[39] Ma Q. (2010). Transcriptional responses to oxidative stress: Pathological and toxicological implications. Pharmacol. Ther..

[40] Finkel T. (2012). Signal transduction by mitochondrial oxidants. J. Biol. Chem..

[41] Wood Z.A., Poole L.B., Karplus P.A. (2003). Peroxiredoxin evolution and the regulation of hydrogen peroxide signaling. Science.

[42] Murphy M.P. (2012). Modulating mitochondrial intracellular location as a redox signal. Sci. Signal..

[43] Dodson M., Darley-Usmar V., Zhang J. (2013). Cellular metabolic and autophagic pathways: Traffic control by redox signaling. Free Radic. Biol. Med..

[44] Al-Mehdi A.B., Pastukh V.M., Swiger B.M., Reed D.J., Patel M.R., Bardwell G.C., Pastukh V.V., Alexeyev M.F., Gillespie M.N. (2012). Perinuclear mitochondrial clustering creates an oxidant-rich nuclear domain required for hypoxia-induced transcription. Sci. Signal..

[45] Hess D.T., Matsumoto A., Kim S.O., Marshall H.E., Stamler J.S. (2005). Protein s-nitrosylation: Purview and parameters. Nat. Rev. Mol. Cell Biol..

[46] Finkel T. (2012). From sulfenylation to sulfhydration: What a thiolate needs to tolerate. Sci. Signal..

[47] Winterbourn C.C., Hampton M.B. (2008). Thiol chemistry and specificity in redox signaling. Free Radic. Biol. Med..

[48] Benhar M., Forrester M.T., Stamler J.S. (2009). Protein denitrosylation: Enzymatic mechanisms and cellular functions. Nat. Rev. Mol. Cell Biol..

[49] Foster M.W., McMahon T.J., Stamler J.S. (2003). S-nitrosylation in health and disease. Trends Mol. Med..

[50] Whalen E.J., Foster M.W., Matsumoto A., Ozawa K., Violin J.D., Que L.G., Nelson C.D., Benhar M., Keys J.R., Rockman H.A. (2007). Regulation of beta-adrenergic receptor signaling by s-nitrosylation of g-protein-coupled receptor kinase 2. Cell.

[51] Nakamura T., Wang L., Wong C.C., Scott F.L., Eckelman B.P., Han X., Tzitzilonis C., Meng F., Gu Z., Holland E.A. (2010). Transnitrosylation of xiap regulates caspase-dependent neuronal cell death. Mol. Cell.

[52] Liu L., Yan Y., Zeng M., Zhang J., Hanes M.A., Ahearn G., McMahon T.J., Dickfeld T., Marshall H.E., Que L.G. (2004). Essential roles of s-nitrosothiols in vascular homeostasis and endotoxic shock. Cell.

[53] Mitchell D.A., Morton S.U., Fernhoff N.B., Marletta M.A. (2007). Thioredoxin is required for s-nitrosation of procaspase-3 and the inhibition of apoptosis in jurkat cells. Proc. Natl. Acad. Sci. USA.

[54] Kim S.F. (2011). The role of nitric oxide in prostaglandin biology; update. Nitric Oxide.

[55] Anand P., Stamler J.S. (2012). Enzymatic mechanisms regulating protein s-nitrosylation: Implications in health and disease. J. Mol. Med..

[56] Church D.F., Pryor W.A. (1985). Free-radical chemistry of cigarette smoke and its toxicological implications. Environ. Health Perspect..

[57] Bedard L., Young M.J., Hall D., Paul T., Ingold K.U. (2001). Quantitative studies on the peroxidation of human low-density lipoprotein initiated by superoxide and by charged and neutral alkylperoxyl radicals. J. Am. Chem. Soc..

[58] Gaschler M.M., Stockwell B.R. (2017). Lipid peroxidation in cell death. Biochem. Biophys. Res. Commun..

[59] Ayala A., Munoz M.F., Arguelles S. (2014). Lipid peroxidation: Production, metabolism, and signaling mechanisms of malondialdehyde and 4-hydroxy-2-nonenal. Oxid. Med. Cell. Longev..

[60] Forman H.J. (2010). Reactive oxygen species and alpha,beta-unsaturated aldehydes as second messengers in signal transduction. Ann. N. Y. Acad. Sci..

[61] Uchida K., Shiraishi M., Naito Y., Torii Y., Nakamura Y., Osawa T. (1999). Activation of stress signaling pathways by the end product of lipid peroxidation. 4-hydroxy-2-nonenal is a potential inducer of intracellular peroxide production. J. Biol. Chem..

[62] Poli G., Schaur R.J., Siems W.G., Leonarduzzi G. (2008). 4-hydroxynonenal: A membrane lipid oxidation product of medicinal interest. Med. Res. Rev..

[63] Zhang H., Court N., Forman H.J. (2007). Submicromolar concentrations of 4-hydroxynonenal induce glutamate cysteine ligase expression in hbe1 cells. Redox Rep..

[64] Kirichenko A., Li L., Morandi M.T., Holian A. (1996). 4-hydroxy-2-nonenal-protein adducts and apoptosis in murine lung cells after acute ozone exposure. Toxicol. Appl. Pharmacol..

[65] Rahman I., van Schadewijk A.A., Crowther A.J., Hiemstra P.S., Stolk J., MacNee W., De Boer W.I. (2002). 4-hydroxy-2-nonenal, a specific lipid peroxidation product, is elevated in lungs of patients with chronic obstructive pulmonary disease. Am. J. Respir. Crit. Care Med..

[66] Droge W. (2002). Free radicals in the physiological control of cell function. Physiol. Rev..

[67] Sung C.C., Hsu Y.C., Chen C.C., Lin Y.F., Wu C.C. (2013). Oxidative stress and nucleic acid oxidation in patients with chronic kidney disease. Oxid. Med. Cell. Longev..

[68] Chelikani P., Fita I., Loewen P.C. (2004). Diversity of structures and properties among catalases. Cell. Mol. Life Sci..

[69] Abate G., Marziano M., Rungratanawanich W., Memo M., Uberti D. (2017). Nutrition and age-ing: Focusing on alzheimer’s disease. Oxid. Med. Cell. Longev..

[70] Tantcheva L.P., Stoeva E.S., Galabov A.S., Braykova A.A., Savov V.M., Mileva M.M. (2003). Effect of vitamin e and vitamin c combination on experimental influenza virus infection. Methods Find. Exp. Clin. Pharmacol..

[71] Stinco C.M., Baroni M.V., Di Paola Naranjo R.D., Wunderlin D.A., Heredia F.J., Meléndez-Martínez A.J., Vicario I.M. (2015). Hydrophilic antioxidant compounds in orange juice from different fruit cultivars: Composition and antioxidant activity evaluated by chemical and cellular based (saccharomyces cerevisiae) assays. J. Food Compos. Anal..

[72] Niki E. (2014). Role of vitamin e as a lipid-soluble peroxyl radical scavenger: In vitro and in vivo evidence. Free Radic. Biol. Med..

[73] Araújo M., Pimentel F.B., Alves R.C., Oliveira M.B.P.P. (2015). Phenolic compounds from olive mill wastes: Health effects, analytical approach and application as food antioxidants. Trends Food Sci. Technol..

[74] Alamed J., Chaiyasit W., McClements D.J., Decker E.A. (2009). Relationships between free radical scavenging and antioxidant activity in foods. J. Agric. Food Chem..

[75] Shahidi F., Ambigaipalan P. (2015). Phenolics and polyphenolics in foods, beverages and spices: Antioxidant activity and health effects—A review. J. Funct. Foods.

[76] Padayatty S.J., Katz A., Wang Y., Eck P., Kwon O., Lee J.H., Chen S., Corpe C., Dutta A., Dutta S.K. (2003). Vitamin c as an antioxidant: Evaluation of its role in disease prevention. J. Am. Coll. Nutr..

[77] Okamoto M., Ueno Y. (2006). Is sudden death with vitamin c deficiency caused by lack of carnitine?. J. Clin. Forensic Med..

[78] Ulrich-Merzenich G., Metzner C., Schiermeyer B., Vetter H. (2002). Vitamin c and vitamin e antagonistically modulate human vascular endothelial and smooth muscle cell DNA synthesis and proliferation. Eur. J. Nutr..

[79] Ulrich-Merzenich G., Metzner C., Bhonde R.R., Malsch G., Schiermeyer B., Vetter H. (2002). Simultaneous isolation of endothelial and smooth muscle cells from human umbilical artery or vein and their growth response to low-density lipoproteins. In Vitro Cell. Dev. Biol. Anim..

[80] Descamps-Latscha B., Drueke T., Witko-Sarsat V. (2001). Dialysis-induced oxidative stress: Biological aspects, clinical consequences, and therapy. Semin. Dial..

[81] Soobrattee M.A., Neergheen V.S., Luximon-Ramma A., Aruoma O.I., Bahorun T. (2005). Phenolics as potential antioxidant therapeutic agents: Mechanism and actions. Mutat. Res..

[82] Hill K.E., McCollum G.W., Boeglin M.E., Burk R.F. (1997). Thioredoxin reductase activity is decreased by selenium deficiency. Biochem. Biophys. Res. Commun..

[83] Adebayo O.L., Adenuga G.A., Sandhir R. (2016). Selenium and zinc protect brain mitochondrial antioxidants and electron transport chain enzymes following postnatal protein malnutrition. Life Sci..

[84] Tomas-Sanchez C., Blanco-Alvarez V.M., Martinez-Fong D., Gonzalez-Barrios J.A., Gonzalez-Vazquez A., Aguilar-Peralta A.K., Torres-Soto M., Soto-Rodriguez G., Limon I.D., Brambila E. (2018). Prophylactic zinc and therapeutic selenium administration increases the antioxidant enzyme activity in the rat temporoparietal cortex and improves memory after a transient hypoxia-ischemia. Oxid. Med. Cell. Longev..

[85] Venugopal R., Jaiswal A.K. (1996). Nrf1 and nrf2 positively and c-fos and fra1 negatively regulate the human antioxidant response element-mediated expression of nad(p)h: Quinone oxidoreductase1 gene. Proc. Natl. Acad. Sci. USA.

[86] Jakel R.J., Townsend J.A., Kraft A.D., Johnson J.A. (2007). Nrf2-mediated protection against 6-hydroxydopamine. Brain Res..

[87] Innamorato N.G., Jazwa A., Rojo A.I., Garcia C., Fernandez-Ruiz J., Grochot-Przeczek A., Stachurska A., Jozkowicz A., Dulak J., Cuadrado A. (2010). Different susceptibility to the parkinson’s toxin mptp in mice lacking the redox master regulator nrf2 or its target gene heme oxygenase-1. PLoS ONE.

[88] Rojo A.I., Innamorato N.G., Martin-Moreno A.M., De Ceballos M.L., Yamamoto M., Cuadrado A. (2010). Nrf2 regulates microglial dynamics and neuroinflammation in experimental parkinson’s disease. Glia.

[89] Vargas M.R., Pehar M., Cassina P., Beckman J.S., Barbeito L. (2006). Increased glutathione biosynthesis by nrf2 activation in astrocytes prevents p75ntr-dependent motor neuron apoptosis. J. Neurochem..

[90] Ramsey C.P., Glass C.A., Montgomery M.B., Lindl K.A., Ritson G.P., Chia L.A., Hamilton R.L., Chu C.T., Jordan-Sciutto K.L. (2007). Expression of nrf2 in neurodegenerative diseases. J. Neuropathol. Exp. Neurol..

[91] Kanninen K., Malm T.M., Jyrkkanen H.K., Goldsteins G., Keksa-Goldsteine V., Tanila H., Yamamoto M., Yla-Herttuala S., Levonen A.L., Koistinaho J. (2008). Nuclear factor erythroid 2-related factor 2 protects against beta amyloid. Mol. Cell. Neurosci..

[92] Kanninen K., Heikkinen R., Malm T., Rolova T., Kuhmonen S., Leinonen H., Yla-Herttuala S., Tanila H., Levonen A.L., Koistinaho M. (2009). Intrahippocampal injection of a lentiviral vector expressing nrf2 improves spatial learning in a mouse model of alzheimer’s disease. Proc. Natl. Acad. Sci. USA.

[93] Chen P.C., Vargas M.R., Pani A.K., Smeyne R.J., Johnson D.A., Kan Y.W., Johnson J.A. (2009). Nrf2-mediated neuroprotection in the mptp mouse model of parkinson’s disease: Critical role for the astrocyte. Proc. Natl. Acad. Sci. USA.

[94] Murakami S., Motohashi H. (2015). Roles of nrf2 in cell proliferation and differentiation. Free Radic. Biol. Med..

[95] Bruns D.R., Drake J.C., Biela L.M., Peelor F.F., Miller B.F., Hamilton K.L. (2015). Nrf2 signaling and the slowed aging phenotype: Evidence from long-lived models. Oxid. Med. Cell. Longev..

[96] Forman H.J., Davies K.J., Ursini F. (2014). How do nutritional antioxidants really work: Nucleophilic tone and para-hormesis versus free radical scavenging in vivo. Free Radic. Biol. Med..

[97] Sun Z., Zhang S., Chan J.Y., Zhang D.D. (2007). Keap1 controls postinduction repression of the nrf2-mediated antioxidant response by escorting nuclear export of nrf2. Mol. Cell. Biol..

[98] Itoh K., Wakabayashi N., Katoh Y., Ishii T., Igarashi K., Engel J.D., Yamamoto M. (1999). Keap1 represses nuclear activation of antioxidant responsive elements by nrf2 through binding to the amino-terminal neh2 domain. Genes Dev..

[99] Kobayashi A., Kang M.I., Okawa H., Ohtsuji M., Zenke Y., Chiba T., Igarashi K., Yamamoto M. (2004). Oxidative stress sensor keap1 functions as an adaptor for cul3-based e3 ligase to regulate proteasomal degradation of nrf2. Mol. Cell. Biol..

[100] Kobayashi A., Kang M.I., Watai Y., Tong K.I., Shibata T., Uchida K., Yamamoto M. (2006). Oxidative and electrophilic stresses activate nrf2 through inhibition of ubiquitination activity of keap1. Mol. Cell. Biol..

[101] Zhang D.D., Lo S.C., Sun Z., Habib G.M., Lieberman M.W., Hannink M. (2005). Ubiquitination of keap1, a btb-kelch substrate adaptor protein for cul3, targets keap1 for degradation by a proteasome-independent pathway. J. Biol. Chem..

[102] McMahon M., Thomas N., Itoh K., Yamamoto M., Hayes J.D. (2006). Dimerization of substrate adaptors can facilitate cullin-mediated ubiquitylation of proteins by a “tethering” mechanism: A two-site interaction model for the nrf2-keap1 complex. J. Biol. Chem..

[103] Baird L., Dinkova-Kostova A.T. (2011). The cytoprotective role of the keap1-nrf2 pathway. Arch. Toxicol..

[104] Yamamoto T., Suzuki T., Kobayashi A., Wakabayashi J., Maher J., Motohashi H., Yamamoto M. (2008). Physiological significance of reactive cysteine residues of keap1 in determining nrf2 activity. Mol. Cell. Biol..

[105] Malhotra D., Portales-Casamar E., Singh A., Srivastava S., Arenillas D., Happel C., Shyr C., Wakabayashi N., Kensler T.W., Wasserman W.W. (2010). Global mapping of binding sites for nrf2 identifies novel targets in cell survival response through chip-seq profiling and network analysis. Nucleic Acids Res..

[106] Tong K.I., Katoh Y., Kusunoki H., Itoh K., Tanaka T., Yamamoto M. (2006). Keap1 recruits neh2 through binding to etge and dlg motifs: Characterization of the two-site molecular recognition model. Mol. Cell. Biol..

[107] Zhang D.D., Hannink M. (2003). Distinct cysteine residues in keap1 are required for keap1-dependent ubiquitination of nrf2 and for stabilization of nrf2 by chemopreventive agents and oxidative stress. Mol. Cell. Biol..

[108] Fourquet S., Guerois R., Biard D., Toledano M.B. (2010). Activation of nrf2 by nitrosative agents and h2o2 involves keap1 disulfide formation. J. Biol. Chem..

[109] Nguyen T., Sherratt P.J., Pickett C.B. (2003). Regulatory mechanisms controlling gene expression mediated by the antioxidant response element. Annu. Rev. Pharmacol. Toxicol..

[110] MacLeod A.K., McMahon M., Plummer S.M., Higgins L.G., Penning T.M., Igarashi K., Hayes J.D. (2009). Characterization of the cancer chemopreventive nrf2-dependent gene battery in human keratinocytes: Demonstration that the keap1-nrf2 pathway, and not the bach1-nrf2 pathway, controls cytoprotection against electrophiles as well as redox-cycling compounds. Carcinogenesis.

[111] Agyeman A.S., Chaerkady R., Shaw P.G., Davidson N.E., Visvanathan K., Pandey A., Kensler T.W. (2012). Transcriptomic and proteomic profiling of keap1 disrupted and sulforaphane-treated human breast epithelial cells reveals common expression profiles. Breast Cancer Res. Treat..

[112] Chorley B.N., Campbell M.R., Wang X., Karaca M., Sambandan D., Bangura F., Xue P., Pi J., Kleeberger S.R., Bell D.A. (2012). Identification of novel nrf2-regulated genes by chip-seq: Influence on retinoid x receptor alpha. Nucleic Acids Res..

[113] Hawkes H.-J.K., Karlenius T.C., Tonissen K.F. (2014). Regulation of the human thioredoxin gene promoter and its key substrates: A study of functional and putative regulatory elements. Biochim. Biophys. Acta Gen. Subj..

[114] Abbas K., Breton J., Planson A.G., Bouton C., Bignon J., Seguin C., Riquier S., Toledano M.B., Drapier J.C. (2011). Nitric oxide activates an nrf2/sulfiredoxin antioxidant pathway in macrophages. Free Radic. Biol. Med..

[115] Jeong W., Bae S.H., Toledano M.B., Rhee S.G. (2012). Role of sulfiredoxin as a regulator of peroxiredoxin function and regulation of its expression. Free Radic. Biol. Med..

[116] Thimmulappa R.K., Mai K.H., Srisuma S., Kensler T.W., Yamamoto M., Biswal S. (2002). Identification of nrf2-regulated genes induced by the chemopreventive agent sulforaphane by oligonucleotide microarray. Cancer Res..

[117] Lee J.M., Calkins M.J., Chan K., Kan Y.W., Johnson J.A. (2003). Identification of the nf-e2-related factor-2-dependent genes conferring protection against oxidative stress in primary cortical astrocytes using oligonucleotide microarray analysis. J. Biol. Chem..

[118] Wu K.C., Cui J.Y., Klaassen C.D. (2011). Beneficial role of nrf2 in regulating nadph generation and consumption. Toxicol. Sci..

[119] Mitsuishi Y., Taguchi K., Kawatani Y., Shibata T., Nukiwa T., Aburatani H., Yamamoto M., Motohashi H. (2012). Nrf2 redirects glucose and glutamine into anabolic pathways in metabolic reprogramming. Cancer Cell.

[120] Singh A., Happel C., Manna S.K., Acquaah-Mensah G., Carrerero J., Kumar S., Nasipuri P., Krausz K.W., Wakabayashi N., Dewi R. (2013). Transcription factor nrf2 regulates mir-1 and mir-206 to drive tumorigenesis. J. Clin. Investig..

[121] Dinkova-Kostova A.T., Holtzclaw W.D., Kensler T.W. (2005). The role of keap1 in cellular protective responses. Chem. Res. Toxicol..

[122] Prestera T., Zhang Y., Spencer S.R., Wilczak C.A., Talalay P. (1993). The electrophile counterattack response: Protection against neoplasia and toxicity. Adv. Enzyme Regul..

[123] Dinkova-Kostova A.T., Massiah M.A., Bozak R.E., Hicks R.J., Talalay P. (2001). Potency of michael reaction acceptors as inducers of enzymes that protect against carcinogenesis depends on their reactivity with sulfhydryl groups. Proc. Natl. Acad. Sci. USA.

[124] Liang L., Gao C., Luo M., Wang W., Zhao C., Zu Y., Efferth T., Fu Y. (2013). Dihydroquercetin (dhq) induced ho-1 and nqo1 expression against oxidative stress through the nrf2-dependent antioxidant pathway. J. Agric. Food Chem..

[125] Kweon M.H., Adhami V.M., Lee J.S., Mukhtar H. (2006). Constitutive overexpression of nrf2-dependent heme oxygenase-1 in a549 cells contributes to resistance to apoptosis induced by epigallocatechin 3-gallate. J. Biol. Chem..

[126] Tang X., Wang H., Fan L., Wu X., Xin A., Ren H., Wang X.J. (2011). Luteolin inhibits nrf2 leading to negative regulation of the nrf2/are pathway and sensitization of human lung carcinoma a549 cells to therapeutic drugs. Free Radic. Biol. Med..

[127] Wruck C.J., Claussen M., Fuhrmann G., Romer L., Schulz A., Pufe T., Waetzig V., Peipp M., Herdegen T., Gotz M.E. (2007). Luteolin protects rat pc12 and c6 cells against mpp+ induced toxicity via an erk dependent keap1-nrf2-are pathway. J. Neural Transm. Suppl..

[128] Eggler A.L., Gay K.A., Mesecar A.D. (2008). Molecular mechanisms of natural products in chemoprevention: Induction of cytoprotective enzymes by nrf2. Mol. Nutr. Food Res..

[129] Hur W., Gray N.S. (2011). Small molecule modulators of antioxidant response pathway. Curr. Opin. Chem. Biol..

[130] Spencer S.R., Xue L.A., Klenz E.M., Talalay P. (1991). The potency of inducers of nad(p)h: (Quinone-acceptor) oxidoreductase parallels their efficiency as substrates for glutathione transferases. Structural and electronic correlations. Biochem. J..

[131] Friling R.S., Bergelson S., Daniel V. (1992). Two adjacent ap-1-like binding sites form the electrophile-responsive element of the murine glutathione s-transferase ya subunit gene. Proc. Natl. Acad. Sci. USA.

[132] Hong F., Sekhar K.R., Freeman M.L., Liebler D.C. (2005). Specific patterns of electrophile adduction trigger keap1 ubiquitination and nrf2 activation. J. Biol. Chem..

[133] Hong F., Freeman M.L., Liebler D.C. (2005). Identification of sensor cysteines in human keap1 modified by the cancer chemopreventive agent sulforaphane. Chem. Res. Toxicol..

[134] Luo Y., Eggler A.L., Liu D., Liu G., Mesecar A.D., van Breemen R.B. (2007). Sites of alkylation of human keap1 by natural chemoprevention agents. J. Am. Soc. Mass Spectrom..

[135] Ohnuma T., Nakayama S., Anan E., Nishiyama T., Ogura K., Hiratsuka A. (2010). Activation of the nrf2/are pathway via s-alkylation of cysteine 151 in the chemopreventive agent-sensor keap1 protein by falcarindiol, a conjugated diacetylene compound. Toxicol. Appl. Pharmacol..

[136] Rachakonda G., Xiong Y., Sekhar K.R., Stamer S.L., Liebler D.C., Freeman M.L. (2008). Covalent modification at cys151 dissociates the electrophile sensor keap1 from the ubiquitin ligase cul3. Chem. Res. Toxicol..

[137] Zhang H., Forman H.J. (2009). Signaling pathways involved in phase ii gene induction by alpha, beta-unsaturated aldehydes. Toxicol. Ind. Health.

[138] Zarkovic N. (2003). 4-hydroxynonenal as a bioactive marker of pathophysiological processes. Mol. Aspects Med..

[139] Sun Q., Spiegelman D., van Dam R.M., Holmes M.D., Malik V.S., Willett W.C., Hu F.B. (2010). White rice, brown rice, and risk of type 2 diabetes in us men and women. Arch. Intern. Med..

[140] Fageria N.K., Baligar V.C. (2003). Upland rice and allelopathy. Commun. Soil Sci. Plant Anal..

[141] Lin Y.T., Pao C.C., Wu S.T., Chang C.Y. (2015). Effect of different germination conditions on antioxidative properties and bioactive compounds of germinated brown rice. BioMed Res. Int..

[142] Cho D.H., Lim S.T. (2016). Germinated brown rice and its bio-functional compounds. Food Chem..

[143] Okarter N., Liu R.H. (2010). Health benefits of whole grain phytochemicals. Crit. Rev. Food Sci. Nutr..

[144] Samyor D., Deka S.C., Das A.B. (2016). Phytochemical and antioxidant profile of pigmented and non-pigmented rice cultivars of Arunachal Pradesh, India. Int. J. Food Prop..

[145] Gul K., Yousuf B., Singh A.K., Singh P., Wani A.A. (2015). Rice bran: Nutritional values and its emerging potential for development of functional food—A review. Bioact. Carbohydr. Diet. Fibre.

[146] Burlando B., Cornara L. (2014). Therapeutic properties of rice constituents and derivatives (*Oryza sativa* L.): A review update. Trends Food Sci. Technol..

[147] Rahman M.A., Hasegawa H., Rahman M.A., Rahman M.M., Miah M.A. (2006). Influence of cooking method on arsenic retention in cooked rice related to dietary exposure. Sci. Total Environ..

[148] Daiponmak W., Senakun C., Siriamornpun S. (2014). Antiglycation capacity and antioxidant activities of different pigmented Thai rice. Int. J. Food Sci. Technol..

[149] Foster-Powell K., Holt S.H., Brand-Miller J.C. (2002). International table of glycemic index and glycemic load values: 2002. Am. J. Clin. Nutr..

[150] Min B., Gu L., McClung A.M., Bergman C.J., Chen M.-H. (2012). Free and bound total phenolic concentrations, antioxidant capacities, and profiles of proanthocyanidins and anthocyanins in whole grain rice (*Oryza sativa* L.) of different bran colours. Food Chem..

[151] Chung I.-M., Kim J.-K., Lee J.-K., Kim S.-H. (2015). Discrimination of geographical origin of rice (*Oryza sativa* L.) by multielement analysis using inductively coupled plasma atomic emission spectroscopy and multivariate analysis. J. Cereal Sci..

[152] Somintara S., Leardkamolkarn V., Suttiarporn P., Mahatheeranont S. (2016). Anti-tumor and immune enhancing activities of rice bran gramisterol on acute myelogenous leukemia. PLoS ONE.

[153] Cicero A.F., Gaddi A. (2001). Rice bran oil and gamma-oryzanol in the treatment of hyperlipoproteinaemias and other conditions. Phytother. Res..

[154] Liu R.H. (2007). Whole grain phytochemicals and health. J. Cereal Sci..

[155] Shao Y., Bao J. (2015). Polyphenols in whole rice grain: Genetic diversity and health benefits. Food Chem..

[156] Sakamoto S., Hayashi T., Hayashi K., Murai F., Hori M., Kimoto K., Murakami K. (2007). Pre-germinated brown rice could enhance maternal mental health and immunity during lactation. Eur. J. Nutr..

[157] Wunjuntuk K., Kettawan A., Rungruang T., Charoenkiatkul S. (2016). Anti-fibrotic and anti-inflammatory effects of parboiled germinated brown rice (oryza sativa ‘kdml 105’) in rats with induced liver fibrosis. J. Funct. Foods.

[158] Boue S.M., Daigle K.W., Chen M.H., Cao H., Heiman M.L. (2016). Antidiabetic potential of purple and red rice (oryza sativa l.) bran extracts. J. Agric. Food. Chem..

[159] Ramarathnam N., Osawa T., Namiki M., Kawakishi S. (1989). Chemical studies on novel rice hull antioxidants. 2. Identification of isovitexin, a c-glycosyl flavonoid. J. Agric. Food Chem..

[160] Ramarathnam N., Osawa T., Namiki M., Kawakishi S. (1989). Studies on changes in fatty acid composition and content of endogenous antioxidants during γ irradiation of rice seeds. J. Am. Oil Chem. Soc..

[161] Hudson E.A., Dinh P.A., Kokubun T., Simmonds M.S., Gescher A. (2000). Characterization of potentially chemopreventive phenols in extracts of brown rice that inhibit the growth of human breast and colon cancer cells. Cancer Epidemiol. Biomark. Prev..

[162] Fardet A., Rock E., Rémésy C. (2008). Is the in vitro antioxidant potential of whole-grain cereals and cereal products well reflected in vivo?. J. Cereal Sci..

[163] Van Hung P. (2016). Phenolic compounds of cereals and their antioxidant capacity. Crit. Rev. Food Sci. Nutr..

[164] Iqbal J., Minhajuddin M., Beg Z.H. (2004). Suppression of diethylnitrosamine and 2-acetylaminofluorene-induced hepatocarcinogenesis in rats by tocotrienol-rich fraction isolated from rice bran oil. Eur. J. Cancer Prev..

[165] Abubakar T., Marikkar N., Salleh A., Azlan A., Jivan M. (2017). Evaluation of brans of different rice varieties for their antioxidative and antihyperglycemic potentials. J. Food Biochem..

[166] Min B., McClung A.M., Chen M.-H. (2011). Phytochemicals and antioxidant capacities in rice brans of different color. J. Food Sci..

[167] Zhang M.-W., Guo B.-J., Zhang R.-F., Chi J.-W., Wei Z.-C., Xu Z.-H., Zhang Y., Tang X.-J. (2006). Separation, purification and identification of antioxidant compositions in black rice. Agric. Sci. China.

[168] Goufo P., Trindade H. (2014). Rice antioxidants: Phenolic acids, flavonoids, anthocyanins, proanthocyanidins, tocopherols, tocotrienols, gamma-oryzanol, and phytic acid. Food Sci. Nutr..

[169] Qureshi A.A., Mo H., Packer L., Peterson D.M. (2000). Isolation and identification of novel tocotrienols from rice bran with hypocholesterolemic, antioxidant, and antitumor properties. J. Agric. Food Chem..

[170] Kim J.-S. (2005). Radical scavenging capacity and antioxidant activity of the e vitamer fraction in rice bran. J. Food Sci..

[171] Afinisha Deepam L.S., Sundaresan A., Arumughan C. (2011). Stability of rice bran oil in terms of oryzanol, tocopherols, tocotrienols and sterols. J. Am. Oil Chem. Soc..

[172] Gong E.S., Luo S.J., Li T., Liu C.M., Zhang G.W., Chen J., Zeng Z.C., Liu R.H. (2017). Phytochemical profiles and antioxidant activity of brown rice varieties. Food Chem..

[173] Tan B.L., Norhaizan M.E. (2017). Scientific evidence of rice by-products for cancer prevention: Chemopreventive properties of waste products from rice milling on carcinogenesis in vitro and in vivo. BioMed Res. Int..

[174] Mitsutoshi I., Eri O., Atsushi K., Akira Y., Ryota K., Masayuki Y., Kazuhiko I., Etsuko N., Kensichi O. (2011). Antioxidant capacities and polyphenol content of colored rice cultivars. Nippon Shokuhin Kagaku Kogaku Kaishi.

[175] Walter M., Marchesan E. (2011). Phenolic compounds and antioxidant activity of rice. Braz. Arch. Biol. Technol..

[176] Terahara N., Saigusa N., Ohba R., Ueda S. (1994). Composition of anthocyanin pigments in aromatic red rice and its wine. Nippon Shokuhin Kogyo Gakkaishi.

[177] Ryu S.N., Park S.Z., Ho C.T. (1998). High performance liquid chromatographic determination of anthocyanin pigments in some varieties of black rice. J. Food Drug Anal..

[178] Abdel-Aal E.-S.M., Young J.C., Rabalski I. (2006). Anthocyanin composition in black, blue, pink, purple, and red cereal grains. J. Agric. Food Chem..

[179] Yoshimura Y., Zaima N., Moriyama T., Kawamura Y. (2012). Different localization patterns of anthocyanin species in the pericarp of black rice revealed by imaging mass spectrometry. PLoS ONE.

[180] Leopoldini M., Russo N., Toscano M. (2011). The molecular basis of working mechanism of natural polyphenolic antioxidants. Food Chem..

[181] Dinkova-Kostova A.T., Abeygunawardana C., Talalay P. (1998). Chemoprotective properties of phenylpropenoids, bis(benzylidene)cycloalkanones, and related michael reaction acceptors: Correlation of potencies as phase 2 enzyme inducers and radical scavengers. J. Med. Chem..

[182] Goffman F., Bergman C. (2004). Rice kernel phenolic content and its relationship with antiradical efficiency. J. Sci. Food Agric..

[183] Yawadio R., Tanimori S., Morita N. (2007). Identification of phenolic compounds isolated from pigmented rices and their aldose reductase inhibitory activities. Food Chem..

[184] Zhang X., Shen Y., Prinyawiwatkul W., King J.M., Xu Z. (2013). Comparison of the activities of hydrophilic anthocyanins and lipophilic tocols in black rice bran against lipid oxidation. Food Chem..

[185] Shih P.H., Yeh C.T., Yen G.C. (2007). Anthocyanins induce the activation of phase ii enzymes through the antioxidant response element pathway against oxidative stress-induced apoptosis. J. Agric. Food Chem..

[186] Lampiasi N., Montana G. (2018). An in vitro inflammation model to study the nrf2 and nf-kappab crosstalk in presence of ferulic acid as modulator. Immunobiology.

[187] Hwang Y.P., Choi J.H., Yun H.J., Han E.H., Kim H.G., Kim J.Y., Park B.H., Khanal T., Choi J.M., Chung Y.C. (2011). Anthocyanins from purple sweet potato attenuate dimethylnitrosamine-induced liver injury in rats by inducing nrf2-mediated antioxidant enzymes and reducing cox-2 and inos expression. Food Chem. Toxicol..

[188] Ichikawa H., Ichiyanagi T., Xu B., Yoshii Y., Nakajima M., Konishi T. (2001). Antioxidant activity of anthocyanin extract from purple black rice. J. Med. Food.

[189] Chen X.Q., Nagao N., Itani T., Irifune K. (2012). Anti-oxidative analysis, and identification and quantification of anthocyanin pigments in different coloured rice. Food Chem..

[190] Xu Z., Godber J.S. (1999). Purification and identification of components of gamma-oryzanol in rice bran oil. J. Agric. Food Chem..

[191] Miller A., Engel K.H. (2006). Content of gamma-oryzanol and composition of steryl ferulates in brown rice (oryza sativa l.) of European origin. J. Agric. Food Chem..

[192] Parrado J., Miramontes E., Jover M., Marquez J.C., Angeles Mejias M., Collantes De Teran L., Absi E., Bautista J. (2003). Prevention of brain protein and lipid oxidation elicited by a water-soluble oryzanol enzymatic extract derived from rice bran. Eur. J. Nutr..

[193] Lerma-García M.J., Herrero-Martínez J.M., Simó-Alfonso E.F., Mendonça C.R.B., Ramis-Ramos G. (2009). Composition, industrial processing and applications of rice bran γ-oryzanol. Food Chem..

[194] Xu Z., Godber J.S. (2001). Antioxidant activities of major components of γ-oryzanol from rice bran using a linoleic acid model. J. Am. Oil Chem. Soc..

[195] Lloyd B.J., Siebenmorgen T.J., Beers K.W. (2000). Effects of commercial processing on antioxidants in rice bran. Cereal Chem..

[196] Metwally A.M., Habib A.M., Khafagy S.M. (1974). Sterols and triterpene alcohols from rice bran oil. Planta Med..

[197] Norton R.A. (1995). Quantitation of steryl ferulate andp-coumarate esters from corn and rice. Lipids.

[198] Walter M., Marchesan E., Massoni P.F.S., da Silva L.P., Sartori G.M.S., Ferreira R.B. (2013). Antioxidant properties of rice grains with light brown, red and black pericarp colors and the effect of processing. Food Res. Int..

[199] Xu Z., Hua N., Godber J.S. (2001). Antioxidant activity of tocopherols, tocotrienols, and gamma-oryzanol components from rice bran against cholesterol oxidation accelerated by 2,2′-azobis(2-methylpropionamidine) dihydrochloride. J. Agric. Food Chem..

[200] Saenjum C., Chaiyasut C., Chansakaow S., Suttajit M., Sirithunyalug B. (2011). Antioxidant and anti-inflammatory activities of gamma-oryzanol rich extracts from thai purple rice bran. J. Med. Plants Res..

[201] Juliano C., Cossu M., Alamanni M.C., Piu L. (2005). Antioxidant activity of gamma-oryzanol: Mechanism of action and its effect on oxidative stability of pharmaceutical oils. Int. J. Pharm..

[202] Kim S.J., Han D., Moon K.D., Rhee J.S. (1995). Measurement of superoxide dismutase-like activity of natural antioxidants. Biosci. Biotechnol. Biochem..

[203] Islam M.S., Yoshida H., Matsuki N., Ono K., Nagasaka R., Ushio H., Guo Y., Hiramatsu T., Hosoya T., Murata T. (2009). Antioxidant, free radical-scavenging, and nf-kappab-inhibitory activities of phytosteryl ferulates: Structure-activity studies. J. Pharmacol. Sci..

[204] Rungratanawanich W., Abate G., Serafini M.M., Guarienti M., Catanzaro M., Marziano M., Memo M., Lanni C., Uberti D. (2018). Characterization of the antioxidant effects of gamma-oryzanol: Involvement of the nrf2 pathway. Oxid. Med. Cell. Longev..

[205] Araujo S.M., de Paula M.T., Poetini M.R., Meichtry L., Bortolotto V.C., Zarzecki M.S., Jesse C.R., Prigol M. (2015). Effectiveness of gamma-oryzanol in reducing neuromotor deficits, dopamine depletion and oxidative stress in a drosophila melanogaster model of parkinson’s disease induced by rotenone. Neurotoxicology.

[206] Ghatak S.B., Panchal S.S. (2012). Anti-diabetic activity of oryzanol and its relationship with the anti-oxidant property. Int. J. Diabetes Dev. Ctries..

[207] Chotimarkorn C., Ushio H. (2008). The effect of trans-ferulic acid and gamma-oryzanol on ethanol-induced liver injury in c57bl mouse. Phytomedicine.

[208] Wilson T.A., Nicolosi R.J., Woolfrey B., Kritchevsky D. (2007). Rice bran oil and oryzanol reduce plasma lipid and lipoprotein cholesterol concentrations and aortic cholesterol ester accumulation to a greater extent than ferulic acid in hypercholesterolemic hamsters. J. Nutr. Biochem..

[209] Accinni R., Rosina M., Bamonti F., Della Noce C., Tonini A., Bernacchi F., Campolo J., Caruso R., Novembrino C., Ghersi L. (2006). Effects of combined dietary supplementation on oxidative and inflammatory status in dyslipidemic subjects. Nutr. Metab. Cardiovasc. Dis..

[210] Niture S.K., Jaiswal A.K. (2012). Nrf2 protein up-regulates antiapoptotic protein bcl-2 and prevents cellular apoptosis. J. Biol. Chem..

[211] Soares M.P., Seldon M.P., Gregoire I.P., Vassilevskaia T., Berberat P.O., Yu J., Tsui T.Y., Bach F.H. (2004). Heme oxygenase-1 modulates the expression of adhesion molecules associated with endothelial cell activation. J. Immunol..

[212] Ahmed S.M., Luo L., Namani A., Wang X.J., Tang X. (2017). Nrf2 signaling pathway: Pivotal roles in inflammation. Biochim. Biophys. Acta.

[213] Pan H., Wang H., Zhu L., Wang X., Cong Z., Sun K., Fan Y. (2013). The involvement of nrf2-are pathway in regulation of apoptosis in human glioblastoma cell u251. Neurol. Res..

[214] Leopoldini M., Marino T., Russo N., Toscano M. (2004). Antioxidant properties of phenolic compounds:  H-atom versus electron transfer mechanism. J. Phys. Chem. A.

[215] Leopoldini M., Marino T., Russo N., Toscano M. (2004). Density functional computations of the energetic and spectroscopic parameters of quercetin and its radicals in the gas phase and in solvent. Theor. Chem. Acc..

[216] Wright J.S., Johnson E.R., DiLabio G.A. (2001). Predicting the activity of phenolic antioxidants:  Theoretical method, analysis of substituent effects, and application to major families of antioxidants. J. Am. Chem. Soc..

[217] Leopoldini M., Pitarch I.P., Russo N., Toscano M. (2004). Structure, conformation, and electronic properties of apigenin, luteolin, and taxifolin antioxidants. A first principle theoretical study. J. Phys. Chem. A.

[218] Chung H.S., Shin J.C. (2007). Characterization of antioxidant alkaloids and phenolic acids from anthocyanin-pigmented rice (*Oryza sativa* cv. Heugjinjubyeo). Food Chem..

[219] Heuberger A.L., Lewis M.R., Chen M.H., Brick M.A., Leach J.E., Ryan E.P. (2010). Metabolomic and functional genomic analyses reveal varietal differences in bioactive compounds of cooked rice. PLoS ONE.

[220] Sirota R., Gibson D., Kohen R. (2015). The role of the catecholic and the electrophilic moieties of caffeic acid in nrf2/keap1 pathway activation in ovarian carcinoma cell lines. Redox Biol..

[221] Talalay P., De Long M.J., Prochaska H.J. (1988). Identification of a common chemical signal regulating the induction of enzymes that protect against chemical carcinogenesis. Proc. Natl. Acad. Sci. USA.

[222] Powers J.C., Asgian J.L., Ekici O.D., James K.E. (2002). Irreversible inhibitors of serine, cysteine, and threonine proteases. Chem. Rev..

[223] Calabrese E.J. (2008). Neuroscience and hormesis: Overview and general findings. Crit. Rev. Toxicol..

[224] Sasaki J., Takada Y., Handa K., Kusuda M., Tanabe Y., Matsunaga A., Arakawa K. (1990). Effects of gamma-oryzanol on serum lipids and apolipoproteins in dyslipidemic schizophrenics receiving major tranquilizers. Clin. Ther..

[225] Berger A., Rein D., Schafer A., Monnard I., Gremaud G., Lambelet P., Bertoli C. (2005). Similar cholesterol-lowering properties of rice bran oil, with varied gamma-oryzanol, in mildly hypercholesterolemic men. Eur. J. Nutr..

[226] Ishihara M. (1984). Effect of gamma-oryzanol on serum lipid peroxide level and clinical symptoms of patients with climacteric disturbances. Asia-Ocean. J. Obstet. Gynaecol..

